# Evidence-Based Use of Undenatured Type II Collagen in the Management of Osteoarthritis in the Indian Subcontinent and Southeast Asia: A Narrative Review and an Expert Opinion

**DOI:** 10.7759/cureus.109411

**Published:** 2026-05-21

**Authors:** Ram Prabhoo, Satish Patel, Indrajit Sardar, Subhash Jangid, Raja V Thirupathy, Ronen Roy

**Affiliations:** 1 Orthopaedics, Mukund Hospital, Mumbai, IND; 2 Orthopaedics, Sarthak Orthopedic Hospital, Ahmedabad, IND; 3 Orthopaedics, Nightingale Hospital, Kolkata, IND; 4 Orthopaedics, Fortis Memorial Research Institute, Haryana, IND; 5 Orthopedics, Visa Medicure Foot Clinic, Chennai, IND; 6 Orthopaedic Surgery, Fortis Hospitals, Kolkata, IND

**Keywords:** indian subcontinent & southeast asia, nutraceuticals, osteoarthritis, uc-ii®, undenatured type ii collagen

## Abstract

Osteoarthritis (OA) is prevalent in the Indian subcontinent and Southeast Asia, causing pain, reduced mobility, and disability. Genetic, lifestyle, and comorbidity factors contribute to earlier onset and more severe disease than in Western populations. Conventional therapies mainly provide symptomatic relief and are limited by adverse effects, underscoring the need for safe, effective adjuncts. Nutraceuticals, particularly undenatured type II collagen, have emerged as promising options. An evidence-based analysis showed that undenatured type II collagen (40 mg/day) improves pain, stiffness, function, and quality of life in early-to-moderate OA (Grades 1-3), with benefits observed within eight weeks, improved compliance, and non-steroidal anti-inflammatory drug-sparing effects. Comparative evidence favors UC-II® (Lonza Greenwood LLC, USA) over other sources, highlighting the importance of product selection. Expert consensus recommends undenatured type II collagen as a nutraceutical adjunct with physiotherapy for long-term use in responders. Future research should include larger trials, biomarker studies, MRI imaging, and real-world data to confirm disease-modifying potential.

## Introduction and background

**Figure 1:**
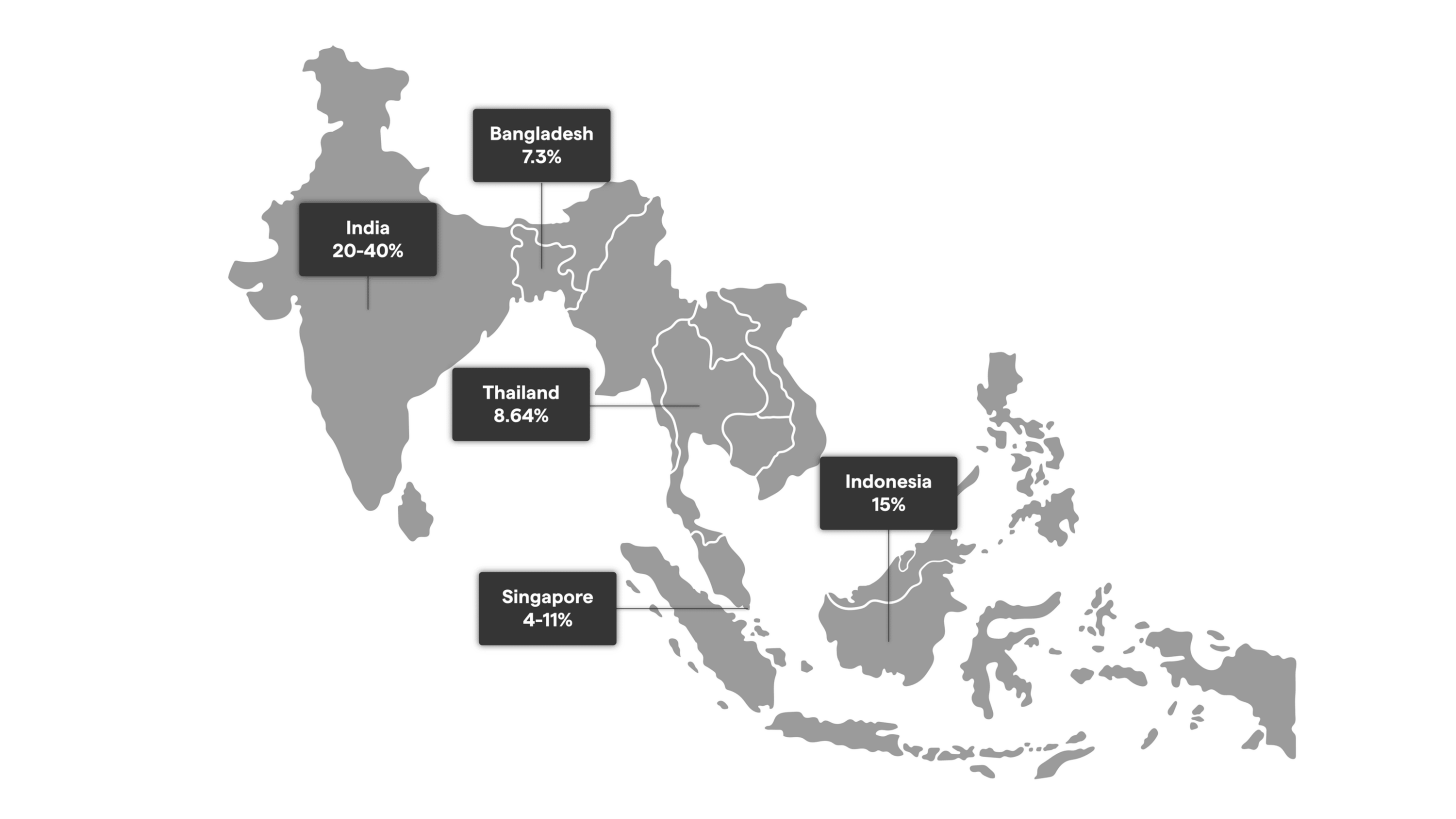
Burden of osteoarthritis across the Indian subcontinent and Southeast Asia

Osteoarthritis (OA) is the most prevalent degenerative joint disease globally and the second most common rheumatologic condition [1]. It causes chronic pain, reduced mobility, and disability, affecting 607 million individuals worldwide, with nearly 46.6 million new cases annually [2]. South Asia ranks among the top three global regions for OA burden, with 16.4% prevalence [3]. Within India, population-based studies show a wide prevalence range of 20%-40%, indicating variation across age, sex, and regional subgroups [4-6]. Other Southeast Asian nations also exhibit high disease rates, including Singapore (4.1%- 11%) [7], Bangladesh (7.3%) [8], Indonesia (15%) [9], and Thailand (8.64%) [10]. These data highlight the OA burden in the Indian subcontinent and Southeast Asia (Figure 1), driven by healthcare access, lifestyle patterns, and genetic predispositions.

Genetic polymorphisms in sterol regulatory element-binding protein 2 (rs2228314) [11], interleukin-6 (rs1800795) [12], and resistin (RETN) [13] in Indian and Pakistani cohorts and growth differentiation factor 5 (rs143383) [14], *HLA-DRB1*, and *HLA-DQB1* in Thai populations [15] are linked to OA risk. These alleles affect cartilage matrix regulation, inflammatory signaling, and immune modulation, predisposing to early-onset and severe OA. The interaction with lifestyle factors (squatting, obesity, diabetes, and vitamin D deficiency) further amplifies susceptibility in South Asians to develop OA [11-15].

Several therapies exist for OA management; however, most primarily provide symptomatic relief rather than altering underlying disease progression or pathophysiology. These include analgesics and non-steroidal anti-inflammatory drugs (NSAIDs), which alleviate pain and inflammation but do not modify the OA pathology. Moreover, prolonged use is linked to adverse gastrointestinal, renal, and cardiovascular complications, as well as hepatic toxicity, hemorrhage, and detrimental impacts on chondrocytes and cartilage matrix formation [16].

In recent years, a rising interest surrounds nutraceutical supplements for OA management, encompassing diverse molecules that reduce inflammation, oxidative stress, pain, and joint stiffness and improve cartilage formation. Chronic nutritional intervention alongside conventional therapy improves OA symptoms more effectively than pharmacological treatment alone [17,18]. Nutraceuticals exert their effect by a multifaceted mechanism of action for OA management by modulating various molecular pathways, including inhibition of tumor necrosis factor-alpha (TNF-α), interleukin-1 beta (IL-1β), matrix metalloproteinases (MMPs), nitric oxide (NO), cyclooxygenase (COX), and caspases [18]. A plethora of nutraceuticals exist in the market today, including glucosamine, chondroitin, minerals and vitamins, curcumin, Boswellia, rosehip, devil’s claw, different types of collagens, ginger, omega-3, 6, and 9, natural eggshell membranes, etc.

A recent meta-analysis of 39 randomized controlled trials (RCTs) demonstrated significant improvements in total Western Ontario and McMaster Universities Osteoarthritis Index (WOMAC) scores with the use of nutraceuticals, including collagen, eggshell membrane, vitamin D, Boswellia, curcumin, ginger, and krill oil [19]. Recent years show considerable interest in nutraceuticals, including symptomatic slow-action drugs for OA (SYSADOA), for example, glucosamine and chondroitin supplementation, to avert damage to the articular cartilage and support the healing process subsequent to the inception of OA [20,21]. Although glucosamine and chondroitin sulfate are the most used agents, the latest American Academy of Orthopaedic Surgeons and American College of Rheumatology guidelines for treating knee OA have given a limited recommendation for using these oral supplements [22,23]. Therefore, there is still an unmet need for clinically proven and rigorously validated nutraceuticals within the SYSADOA category to ensure effective and reliable management of OA.

Collagen constitutes the primary structural protein of articular cartilage, and its degradation plays a central role in knee OA progression. Disruption of the collagen network compromises cartilage integrity and joint function. Several RCTs and meta-analyses suggest that oral collagen supplementation may improve symptoms and functional outcomes in patients with OA [24-27].

Collagen supplements, broadly classified into hydrolyzed collagen and undenatured type II collagen, differ in their biological effects, as variations in type, structure, and processing significantly influence their mechanism of action and clinical efficacy in OA management. Hydrolyzed collagen contains small peptides derived after original collagen structure disintegration, serving as precursors for cartilage extracellular matrix synthesis, whereas undenatured type II collagen acts via an immunomodulatory mechanism, inducing oral tolerance and reducing joint inflammation. Effective dosages differ markedly: hydrolyzed collagen requires relatively high intake (2-10 g/day) to achieve therapeutic benefits, whereas undenatured type II collagen is effective at a much lower dose (~40 mg/day) [25-27].

**Figure 2:**
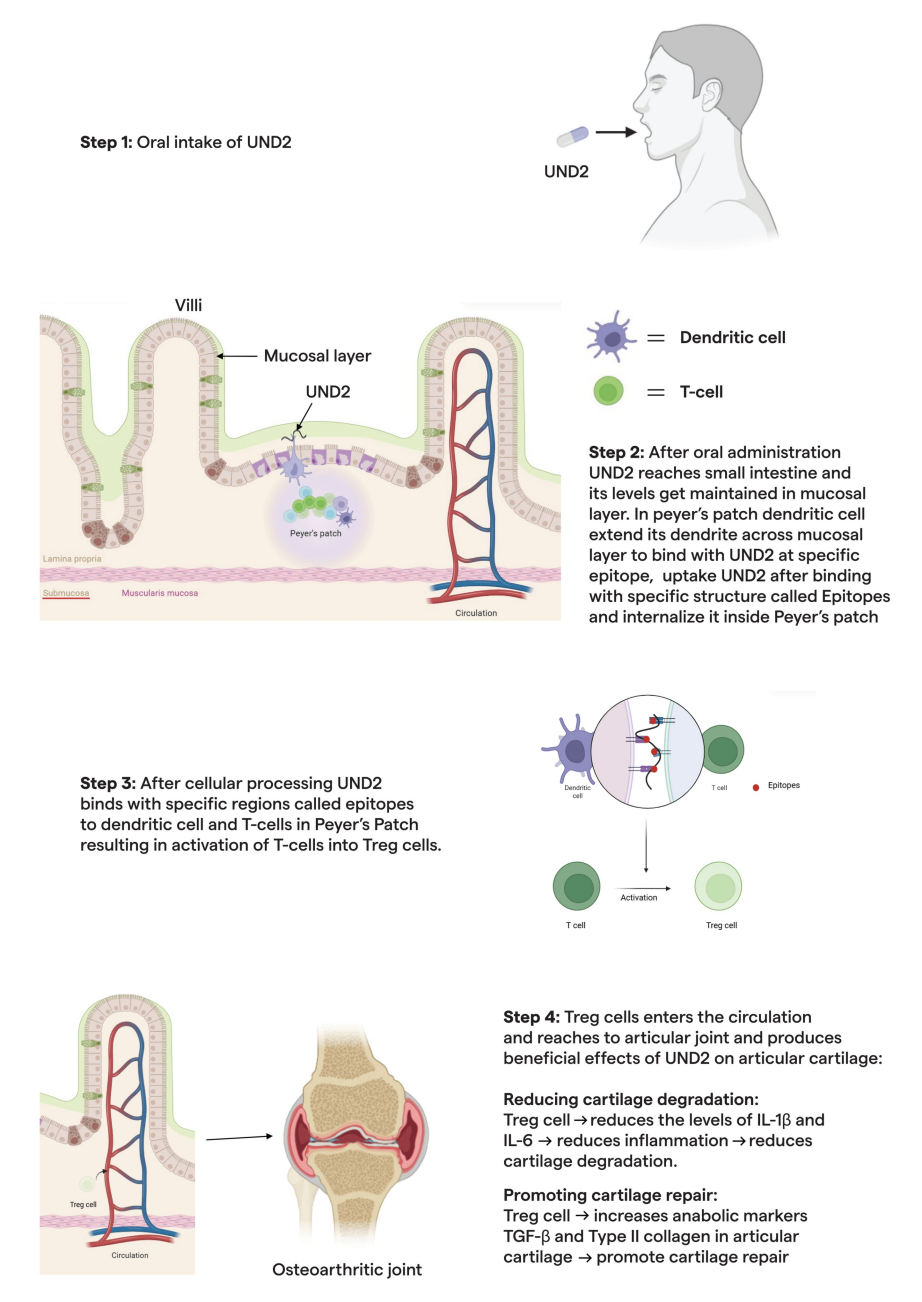
Mechanism of action of undenatured type II collagen IL, interleukin; TGF, transforming growth factor; UND2, undenatured type II collagen. This figure has not been generated using any artificial intelligence tools. The figure was created using BioRender software.

Undenatured type II collagen uniquely stands out among collagen supplements due to its structural integrity, immunomodulatory mechanism, and high patient acceptability. When administered orally (Figure 2), undenatured type II collagen survives gastric digestion and reaches the small intestine intact, where specialized lymphoid tissues called Peyer’s patches reside, containing dendritic cells and T cells beneath a protective mucosal layer, essential for inducing immune tolerance. Dendritic cells capture undenatured type II collagen via its intact epitopes, internalize it, and present it to T cells through the major histocompatibility complex. This process activates T cells into regulatory T cells (Treg), which subsequently migrate via the bloodstream to the joints. In the joint environment, Treg cells suppress inflammation by increasing IL-10 and reducing pro-inflammatory cytokines such as IL-1β and IL-6, while enhancing anabolic factors, including transforming growth factor-beta and type II collagen. This dual action prevents cartilage degradation and promotes cartilage repair through fibrocartilage formation [28-30]. The structural specificity of undenatured type II collagen epitopes is critical: intact epitopes are required both for dendritic cell capture and T-cell binding. Hydrolyzed or denatured collagen lacking these epitopes cannot induce Treg activation, highlighting that epitope integrity is indispensable for oral tolerance and the joint health benefits of undenatured type II collagen [31-33].

## Review

Need for expert opinion

Despite growing clinical and mechanistic evidence for collagen supplementation in OA, several gaps and challenges remain in translating this knowledge into clinical practice, especially in the Indian subcontinent and Southeast Asia.

South Asian populations demonstrate earlier onset, higher prevalence, and greater disease severity compared to Western cohorts. In addition, cultural and occupational risk factors (squatting and manual labor) and comorbidities (obesity, diabetes, and vitamin D deficiency) contribute to a distinct OA phenotype. Consequently, the extrapolation of Western trial data may not always be appropriate for Indian and Southeast Asian contexts [34]. Additionally, although undenatured type II collagen can improve WOMAC, visual analog scale (VAS), and functional mobility outcomes, clinicians frequently seek clarity on whether it is best used. This paper answers clinical questions such as whether it can be used as a first-line adjunct in OA, up to which grade of OA undenatured type II collagen can be prescribed, and does long-term maintenance therapy delay disease progression and surgery.

Comparative evidence among different undenatured type II collagen available in the region remains limited, with studies demonstrating considerable heterogeneity in terms of study design, endpoints, and population characteristics. This variability hinders clear differentiation in these products’ efficacy and safety profiles. To address this, a regional expert advisory board was essential to critically evaluate the available literature and its clinical relevance among Indian subcontinent and Southeast Asian patients. This document aimed to provide evidence-informed recommendations on the appropriate use, dosing, duration, and clinical positioning within treatment protocols; to review clinical data pertaining to various undenatured type II collagens available in these regions; to identify existing research gaps; and to outline priorities for future investigations.

This expert opinion document reflects not only the available scientific literature but also the collective expertise of leading clinicians from India, addressing practical considerations to consider while using undenatured type II collagen in promoting patient care, and can help in clinical decision-making for OA management.

Methodology

Literature Search

A comprehensive literature search was conducted using the PubMed, Scopus, and Google Scholar databases, covering all available records from database inception to July 2025, and was restricted to the English language only. The search was performed using a combination of free-text keywords and controlled vocabulary (MeSH/Emtree terms). The detailed PubMed search string was as follows: (“Osteoarthritis”[MeSH Terms] OR “osteoarthritis”[Title/Abstract] OR “knee osteoarthritis”[Title/Abstract] OR “hip osteoarthritis”[Title/Abstract]) AND (“Collagen Type II”[MeSH Terms] OR “undenatured type II collagen”[Title/Abstract] OR “native type II collagen”[Title/Abstract]) AND (“Treatment Outcome”[MeSH Terms] OR “efficacy”[Title/Abstract] OR “safety”[Title/Abstract] OR “randomized controlled trial”[Publication Type] OR “clinical trial”[Title/Abstract] OR “real-world evidence”[Title/Abstract]). Truncations (*) were used at appropriate places to ensure all variations of search terms were included.

Studies were included based on predefined eligibility criteria. Eligible studies comprised human clinical investigations, including RCTs and observational studies, enrolling patients diagnosed with OA. Studies conducted only in the Indian subcontinent and Southeast Asia were included. Only studies reporting clinically relevant efficacy outcomes, such as the WOMAC, VAS, or validated functional assessment tools, were considered. Exclusion criteria encompassed animal or in vitro studies, case reports, editorials, review articles, conference abstracts without full text, and articles not reporting clinical outcomes. Studies that did not clearly specify the form or type of collagen evaluated were also excluded to ensure methodological consistency. To ensure comprehensive coverage, reference lists of relevant reviews and included studies were manually screened for additional eligible studies.

An advisory board meeting was convened virtually on August 09, 2025. The meeting brought together a group of six expert orthopedic surgeons having decades of experience in this field across the region to evaluate the current evidence on undenatured type II collagen and its role in OA management.

The document was developed through a structured, multistep process that began with context setting on the burden of OA in the Indian subcontinent and Southeast Asia, and a defined objective to evaluate undenatured type II collagen for the management of OA in an unbiased manner. This was followed by evaluating clinical data available on different types of undenatured type II collagen available in the region. An interactive panel discussion then integrated expert clinical experiences, addressing positioning of undenatured type II collagen across various OA grades, comparative efficacy of different types of undenatured type II collagen available in the Indian subcontinent and Southeast Asian region, NSAID-sparing potential, and use in comorbid populations. Insights were synthesized into practice-oriented expert recommendations.

Results

**Figure 3:**
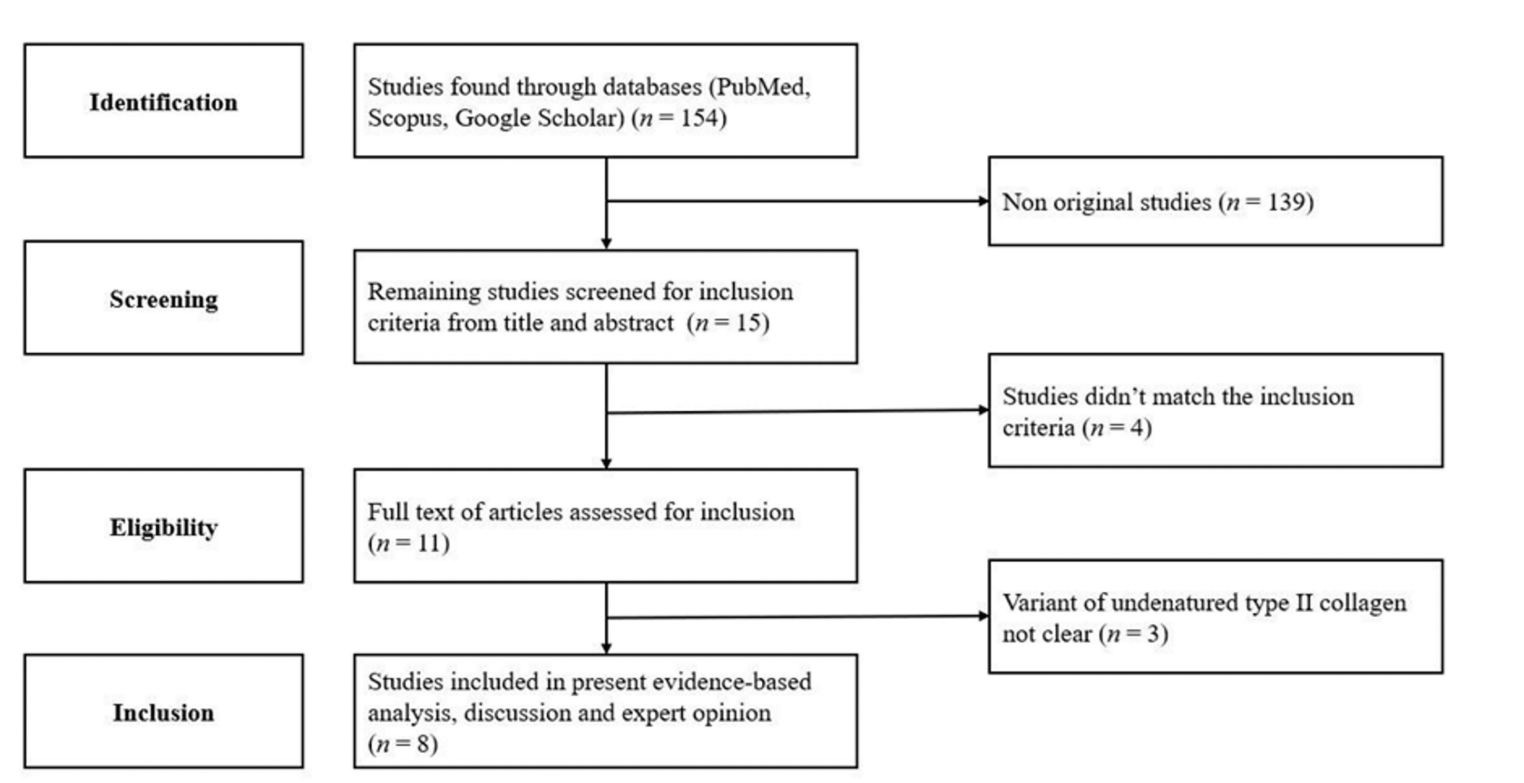
Details of search strategy and its outcomes

A preliminary search retrieved 154 records from all databases combined. After removing duplicates and studies deemed irrelevant to our research scope, 15 studies were identified for further evaluation (Figure 3). Following the exclusion of articles that did not meet the inclusion criteria, 11 studies remained. Of these, three were excluded due to a lack of clarity regarding the variant of undenatured type II collagen examined [35-37]. After a thorough full-text assessment, eight studies were ultimately included in the present review.

All included studies evaluated the role of undenatured type II collagen in the management of OA as monotherapy in the Indian subcontinent and Southeast Asian populations. Among the selected studies, four were randomized, double-blind, placebo-controlled [38-41], while the other four included a single-arm interventional study [42], a real-world observational study [43], a randomized controlled study [44], and a prospective RCT [45]. Syndicated third party data identified the following types of undenatured type II collagen available in the Indian subcontinent and Southeast Asian markets: UC-II® (Lonza Greenwood LLC, USA), Unstergen® (Titan Biotech Ltd., India), Native CT-II® (HS Nutra Co. Ltd., China), Collavant n2® (Bioiberica, Spain), Carticol® (Ten Kate Holding B.V., The Netherlands), native type II collagen (Nitta Gelatin India Limited, India), Smarticol® (Ten Kate Holding B.V., The Netherlands), and Nuvicol® (Ten Kate Holding B.V., The Netherlands). A summary of the included studies is mentioned in Table 1.

**Table 1:** Clinical evidence on the role of undenatured type II collagen in the management of OA in the Indian subcontinent and Southeast Asian populations BMI, body mass index; EQ-5D VAS, EuroQol 5-dimension visual analog scale; G+C, glucosamine + chondroitin; LFI, Lequesne functional index; M:F, male to female ratio in numbers; NRS, numerical rating scale; T, treatment group (undenatured type II collagen group); VAS, visual analog scale; WOMAC, Western Ontario and McMaster Universities Osteoarthritis Index.

Parameters	Lugo et al. [38]	Tjandra et al. [39]	Mehra et al. [43]	Azeem et al. [42]	Sree [45]	Singh et al. [44]	Luo et al. [40]	Sriraam et al. [41]
Design	Randomized double-blind placebo-controlled trial	Randomized double-blind placebo-controlled trial	Real-world observational study	Single-arm interventional study	Prospective randomized controlled study	Randomized controlled study	Randomized double-blind placebo-controlled trial	Randomized double-blind placebo-controlled trial
Sample size	191	101	226	100	70	80	101	48
Sample age (years)	40-75	40-75	28-79	45-65	45-65	41-60	40-65	30-65
Male:female ratio	M:F (in no.): placebo: 28:30; G+C: 28:37; T: 33:30	M:F (in no.): placebo: 3:46; T: 7:43	M:F (in no.): T: 136:155	M:F (in no.): 43:57	M:F (in no.): 20:50	M:F (in no.): 30:50	>70% females	M:F (in no.): 28:17
BMI range	18-30 kg/m^2^	18-30 kg/m²	26.6±3.7 kg/m²	24-30 kg/m²	Normal: 14%; overweight: 46%; obese: 40%	Not reported	18.5-29.9 kg/m² (mean: ~25 across groups)	T group: 27.08±4.16 kg/m²; placebo: 26.94±3.16 kg/m²
OA grade	2 to 3	2 to 3	2 to 3	2 to 3	1 to 3	2 to 3	2 to 3	Not available
Intervention	Placebo G+C: 1500 mg + 1200 mg/day; T: 40 mg/day	Placebo T: 40 mg/day	T: 40 mg/day	T: 40 mg/day	T: 40 mg/day + etodolac; comparator group: etodolac + G (1500 mg)	T: 40 mg/day; comparator group: glucosamine (1500 mg/day)	Placebo G+C = 1500 mg + 1200 mg/day; T: 40 mg/day	Placebo T: 40 mg/day
Type of undenatured type II collagen used	UC-II®	UC-II®	UC-II®	UC-II®	UC-II®	UC-II®	Native CT-II®	Unstergen®
Duration	Six months	Three months	Three months	Four months	Six months	Three months	Three months	Three months
Location	India	Indonesia	India	India	India	India	India	India
Results	The T group demonstrated a significant reduction in overall WOMAC score compared with placebo (p=0.002) and G+C (p=0.04) at day 180, with improvements in pain, stiffness, and function subscales. The T group showed a significant reduction in mean VAS (p=0.025 vs. G+C) and LFI scores (p=0.009 vs. placebo).	The T group led to a significant reduction in the WOMAC score as early as Day 7 (p<0.05), with an 81.6% reduction in the T group vs. 19.2% in the placebo group. After Day 90, improvement in the T group vs. the placebo group in total LFI was 75.8% vs. 7.8% (p<0.01), and in VAS was 67.9% vs. 12.2% (p<0.01).	The T group led to a significant reduction in the WOMAC score from baseline as early as Day 30 (p<0.001) by 13.59%. A significant reduction in VAS score was also observed as early as Day 30 (p<0.001) by 14.28%. By Day 90, WOMAC scores improved by 40.11% and VAS scores decreased by 52.26%.	All outcomes improved significantly at the end of four months: pain (p=0.0005), stiffness (p=0.004), physical function (p=0.002), VAS score (p=0.002), and LFI score (p=0.008).	T group reduced WOMAC scores by 41% after 90 days, compared with 20% in the comparator group (p<0.0001). VAS scores decreased by 43% in the T group compared with 28% in the comparator group after 90 days (p<0.0001).	WOMAC scores showed a significant reduction within both groups over three months (p<0.0001). VAS scores showed a significant reduction in both groups over three months (p<0.0001).	T group leads a significant reduction in WOMAC total score as early as Week 4 (p=0.0036), with continued improvement through Week 12. The effect was equivalent to the G+C combination. The T group had a significantly improved EQ-5D VAS score compared with placebo at Week 12 (p=0.0003).	The T group showed a 20.39% reduction in WOMAC scores compared with the placebo group (p=0.000). Pain NRS scores decreased by 37.77% in the T group vs 8.70% in the placebo group (p=0.000). Subjects in the T group reported an improved quality of life compared with the placebo group (p<0.05).
Tolerability and safety	Good safety profile with no adverse effects							

Effect of Undenatured Type II Collagen in Improving OA Symptoms

Evidence: Clinical studies (Table 1) showed that undenatured type II collagen has consistently demonstrated good efficacy and safety in the management of OA in the Indian subcontinent and Southeast Asian populations. Evidence spans improvements in pain, mobility, and joint function, along with NSAID-sparing benefits, better quality of life, and a favorable safety profile.

Expert opinion: Undenatured type II collagen was found to be a well-studied and clinically validated option for the management of OA as an adjuvant therapy in current clinical practice. Experts noted an onset of benefits typically within four to six weeks, with progressive improvements in pain and mobility. It was regarded as particularly effective in Grade 2-3 OA, where it helped reduce pain and maintain mobility. The experts mentioned that it may have an NSAID-sparing role (reduction in NSAID dose). However, panelists observed differences in the efficacy of various types of undenatured type II collagen available in the Indian subcontinent and Southeast Asian region. They observed that UC-II® ingredient can be considered the preferred undenatured type II collagen to be used as an adjunct for OA management due to the larger body of clinical evidence available on it. It is also found to have superior performance on WOMAC scores compared with other commercially available undenatured type II collagen in this region.

Selection Criteria of Undenatured Type II Collagen

Evidence: The clinical effectiveness of collagen supplements is highly dependent on structural preservation, dose, regimen, and patient compliance. Several selection parameters are critical for clinicians when choosing between the available variants in the collagen category.

Dose and convenience: Undenatured type II collagen requires a once-daily dose of 40 mg, a significant advantage in terms of higher patient compliance [38-45].

Efficacy parameters: Undenatured type II collagen has been reported to be effective in the management of OA, showing improvements in WOMAC, VAS, and functional outcomes [38-45]. However, efficacy varies among different types of undenatured type II collagen available in the Indian subcontinent and Southeast Asian region. For example, UC-II® ingredient has been shown to be superior to the combination of glucosamine (1500 mg) and chondroitin (1200 mg) [38], whereas Native CT-II® was found to be equivalent to the same glucosamine-chondroitin combination (in terms of effect on total WOMAC scores) [40], when administered at the same dose, for the same duration, and in a comparable population with similar OA severity (Grade 2-3) and genotype. Furthermore, as discussed earlier, different types of undenatured type II collagen available in region of the Indian subcontinent and Southeast Asia also differ in terms of effect size on total WOMAC scores [38,40,41].

Compliance: Undenatured type II collagen showed high patient compliance due to low dose [38-45].

Safety and tolerability: Undenatured type II collagen is consistently reported as safe and well tolerated across clinical trials, with no significant gastrointestinal, renal, or hepatic adverse effects [38-45].

Expert opinion: Expert panel acknowledged that undenatured type II collagen is supported by validated clinical evidence for use in the management of OA as an adjuvant therapy, particularly with a once-daily low-dose regimen of 40 mg, which is associated with improved patient compliance. Based on collective clinical experience, the experts noted that once-daily oral formulations are highly acceptable to patients, especially elderly individuals with polypharmacy, as the low pill burden and observable onset of benefits within approximately eight weeks facilitate sustained long-term adherence. The panel recommended the use of undenatured type II collagen in patients with early-to-moderate OA (Grades 1-3) to alleviate pain and preserve joint function. Based on their clinical experience, the panel suggested that supplementation of undenatured type II collagen can potentially delay disease progression. It was further considered a suitable option for patients with common comorbidities, including diabetes, hypertension, and renal impairment, offering a safer alternative to NSAIDs for long-term usage, particularly in individuals at increased risk of NSAID-related adverse effects. However, the panel emphasized that all undenatured type II collagens should not be considered interchangeably; for example, the UC-II® ingredient from Lonza Greenwood LLC should not be regarded as interchangeable with different types of undenatured type II collagen available in the Indian subcontinent and Southeast Asia, as current evidence suggests that UC-II® is more extensively clinically validated than other undenatured type II collagen variants available in the region.

Comparison of Different Undenatured Type II Collagen Available in the Indian Subcontinent and Southeast Asian Region

Evidence: Several undenatured type II collagen are available in the Indian subcontinent and Southeast Asia, though the UC-II® ingredient remains the most extensively investigated. Comparative data highlight significant variability clinical endpoints and strength of evidence.

UC-II® ingredient has a total of six published studies (five conducted in India and one in Jakarta, Indonesia). In contrast, Unstergen® and Native CT-II® each have only one clinical study. Other undenatured type II collagen available in the considered regional market, viz. Collavant n2® and Carticol®. Native type II collagen, Smarticol®, and Nuvicol® have no clinical studies reported in the Indian subcontinent or Southeast Asian populations. The UC-II® ingredient has been evaluated in a total of 514 volunteers, whereas all other undenatured type II collagen available in the considered geographical region combined have been studied in only 61 volunteers collectively.

A significant difference in effect size was observed among different undenatured type II collagen available in the Indian subcontinent and Southeast Asian region when compared at the same time points [38,40,41]. At the 90-day time point, THE UC-II® ingredient demonstrated a 48.5-point reduction in the total WOMAC score, whereas Native CT-II® and Unstergen® showed reductions of 32.47 and 12.19 points, respectively, when evaluated in patients with similar OA severity grades and comparable genotypic populations.

Expert opinion: Based on available evidence, the UC-II® ingredient is the only undenatured type II collagen supported by strong, reproducible clinical data across multiple studies in the Indian subcontinent and Southeast Asian populations. Native CT-II® and Unstergen® have demonstrated some efficacy; however, experts emphasized that their data remain limited in scale, duration, and robustness, requiring further clinical validation. Panelists highlighted the need to clearly differentiate the UC-II® ingredient from other undenatured type II collagen available in the region, as not all undenatured type II collagen available delivers the same clinical outcomes when compared on total WOMAC scores within similar OA severity levels and comparable population pools. Experts further noted that, based on available evidence, the structural complexity of undenatured type II collagen is a key differentiating factor. Preservation of specific epitopes within the undenatured type II collagen structure is critical for the development of oral tolerance [31-33]. It is scientifically inappropriate to extrapolate clinical data from UC-II® ingredient to other undenatured type II collagen available in the region, as molecular equivalence has not been established, and clinical outcomes differ.

Clinicians agreed that, based on the current body of evidence, the UC-II® ingredient should serve as the reference standard for undenatured type II collagen in OA management, while for other types of undenatured type II collagen, more data need to validate the clinical outcomes since non-equivalency is evident from the available clinical data.

Position in Therapy

The current management of OA involves a multimodal approach, combining lifestyle modification, pharmacological therapy, and nutraceutical support, with undenatured type II collagen emerging as a promising option within this continuum. As summarized in Table 2, the expert panel endorsed undenatured type II collagen as a preferred first-line nutraceutical in early OA (up to Grade 3) due to strong evidence, convenience, and excellent tolerability. In OA (up to Grade 3), it is recommended as an adjunct rather than a substitute for pharmacological therapy, with its NSAID-sparing effect highlighted as a key clinical advantage. Unlike NSAIDs or steroids, it is suitable for long-term use, making it valuable for chronic management. Clinically, it is often introduced when patients fail to respond adequately to glucosamine or chondroitin, with reported improvements in satisfaction and functional outcomes post-switch. As per the authors’ clinical experience, while it cannot replace arthroplasty in advanced OA, it helps maintain functional independence, provides symptomatic relief, and may delay the need for total knee replacement by up to five years. Further in clinical experience, undenatured type II collagen demonstrated adjunctive value with disease-modifying antirheumatic drugs in rheumatoid arthritis by reducing swelling and pain, and has been proven effective in chondromalacia up to Grade 3, particularly in women over 30 with patellar cartilage involvement.

**Table 2:** Expert recommendations on the use of undenatured type II collagen in OA management G+C, glucosamine + chondroitin; LSI, Lequesne severity index; NSAID, non-steroidal anti-inflammatory drug; OA, osteoarthritis; VAS, visual analog scale; WOMAC, Western Ontario and McMaster Universities Osteoarthritis Index.

Parameter	Expert recommendation
Clinical use	1. Initiate undenatured type II collagen at a 40 mg once daily dose early in OA Grade 1-3 as a first-line nutraceutical adjunct with physiotherapy and weight management.
	2. Evaluate outcomes at three to six months using WOMAC, VAS, or LSI; benefits may appear as early as eight weeks.
	3. Continue as long-term therapy in responders, given its excellent safety profile.
Therapeutic positioning	4. Employ undenatured type II collagen as an NSAID-sparing adjunct in symptomatic OA, allowing reduction in analgesic/NSAID burden.
	5. Use in OA (up to Grade 3) as supportive therapy until surgery.
	6. Position undenatured type II collagen within multimodal OA care, complementing short NSAID courses, corticosteroid injections, or hyaluronic acid where indicated.
Product differentiation	7. UC-II® ingredient should not be considered interchangeable with other undenatured type II collagen available in the region, as no equivalency data exist, and clinical outcomes vary across different undenatured type II collagens. UC-II® supplementation is supported by multiple randomized controlled trials and real-world evidence.
	8. Undenatured type II collagen is preferred over hydrolyzed collagen in clinical practice due to its low dose, and it is better than the glucosamine/chondroitin (G+C) combination (using UC-II^®^), particularly in non-responders to G+C.
Patient selection	9. Favorable in mild-moderate OA (up to Grade 3), patients with NSAID contraindications, or the elderly with polypharmacy due to low dose and high compliance.
	10. Safe in patients with comorbidities (e.g., diabetes, hypertension, and renal insufficiency).
	11. Extend undenatured type II collagen use to relevant peri-procedural contexts (e.g., after arthroscopy) and in select non-OA joint health scenarios (e.g., chondromalacia). Experts report supportive experience while acknowledging the need for more rigorous data in these indications.

Future Directions

The expert panel emphasized the need for longitudinal studies, particularly long-term RCTs (>1 year), to assess whether undenatured type II collagen can truly delay disease progression. The group also highlighted an urgent need for head-to-head comparative data to evaluate the UC-II® ingredient against other undenatured type II collagen available in the region under identical conditions, as equivalency among them is not scientifically substantiated. Panelists further encouraged mechanistic and biomarker studies, including the use of cartilage degradation biomarkers and imaging modalities such as MRI and ultrasound, to strengthen evidence for the disease-modifying potential of undenatured type II collagen. Experts additionally suggested patient subgroup analyses by stratifying outcomes based on OA severity, age, metabolic comorbidities, and genetic background to enable more tailored treatment recommendations. The experts also proposed that future clinical guidelines in the Indian subcontinent and Southeast Asia should formally incorporate undenatured type II collagen, based on evolving evidence. Finally, clinicians called for the generation of real-world evidence through large-scale registry data from South Asia to capture long-term outcomes, adherence, and NSAID-sparing benefits in routine clinical practice.

## Conclusions

Undenatured type II collagen exhibits a unique immunomodulatory mechanism that supports oral tolerance, reduces joint inflammation, and promotes cartilage repair, with demonstrated improvements in pain, function, and quality of life. Clinical evidence supports the efficacy and safety of undenatured type II collagen for the management of OA symptoms, in terms of WOMAC, VAS, and functional indices, with a favorable safety profile, a low dose requirement (40 mg/day), high patient compliance, and NSAID-sparing benefits. Comparative evidence indicates that not all undenatured type II collagen available in the region is equivalent, and UC-II® should be considered the reference standard based on multiple RCTs and real-world data, with a clear differential effect with respect to other undenatured type II collagen available in the region. Experts recommend early initiation of undenatured type II collagen in Grades 1-3 OA as a first-line nutraceutical adjunct alongside lifestyle interventions and physiotherapy, with continued long-term use in responders. Its role extends to reducing analgesic dependence, supporting peri-procedural care, and complementing multimodal OA therapy, while offering a safe option for patients with comorbidities.
